# Appropriate scaling approach for evaluating peak VO_2_ development in Southern Chinese 8 to 16 years old

**DOI:** 10.1371/journal.pone.0213674

**Published:** 2019-03-12

**Authors:** Clare C. W. Yu, Ali M. McManus, Chun T. Au, Hung K. So, Adrienne Chan, Rita Y. T. Sung, Albert M. Li

**Affiliations:** 1 Department of Pediatrics, Prince of Wales Hospital, The Chinese University of Hong Kong, Hong Kong SAR, Hong Kong; 2 School of Health and Exercise Sciences, The University of British Columbia, Kelowna, Canada; 3 Department of Paediatrics and Adolescent Medicine, Queen Mary Hospital, The University of Hong Kong, Hong Kong SAR, Hong Kong; Ospedale del Cuore G Pasquinucci Fondazione Toscana Gabriele Monasterio di Massa, ITALY

## Abstract

**Objective:**

To investigate scaling approaches for evaluating the development of peak VO_2_ and improving the identification of low cardiopulmonary fitness in Southern Chinese children and adolescents.

**Methods:**

Nine hundred and twenty Chinese children and adolescents (8 to 16 years) underwent graded cardiopulmonary exercise test on a treadmill until volitional exhaustion. Peak VO_2_ was corrected for the effects of body mass by ratio or allometric scaling. Z score equations for predicting peak VO_2_ were developed. Correlations between scaled peak VO_2_, z scores, body size and age were tested to examine the effectiveness of the approach.

**Results:**

Eight hundred and fifty-two participants (48% male) were included in the analyses. Absolute peak VO_2_ significantly increased with age in both sexes (both *P<*0.05), while ratio-scaled peak VO_2_ increased only in males (*P<*0.05). Allometrically scaled peak VO_2_ increased from 11 years in both sexes, plateauing by 12 years in girls and continuing to rise until 15 years in boys. Allometically scaled peak VO_2_ was not correlated with body mass, but remained correlated with height and age in all but the older girls. Peak VO_2_ z score was not correlated with body mass, height or age.

**Conclusions:**

Absolute and allometric scaled peak VO_2_ values are provided for Hong Kong Chinese children and adolescents by age and sex. Peak VO_2_ z scores improve the evaluation of cardiopulmonary fitness, allowing comparisons across ages and sex and will likely provide a better metric for tracking change over time in children and adolescents, regardless of body size and age.

## Introduction

It is well established that adequate aerobic fitness is associated with reduced risk of all-cause mortality and chronic diseases across the lifespan[1–5]. Peak oxygen uptake (peak VO_2_) is widely recognized as one of the best indicators of aerobic fitness in the child, providing a composite measure of the pulmonary, cardiovascular, and hematological components of oxygen delivery and oxygen utilization in the exercising muscles.

In clinical settings, peak VO_2_ has been used as a predictor of mortality and of hospital admissions [6–8], an indicator of the severity of functional impairment and for tracking responses to intervention [9]. Despite the proven usefulness of peak VO_2_, achieving quality and consistency of data in children remains a challenge. Peak VO_2_ is developmentally divergent, varying with age, maturity and sex, and is highly correlated with body size and composition [10, 11]. It has been convention to scale peak VO_2_ by simply dividing absolute peak VO_2_ (mL. min^-1^) by body mass (ratio scaling). This however, results in a different pattern of development in peak VO_2_ in comparison to absolute peak VO_2_, with absolute peak VO_2_ in boys increasing with age, but ratio-scaled peak VO_2_ remaining unchanged [12]. Similarly in girls, increases in absolute peak VO_2_ are noted with increasing age until a levelling off in puberty, but ratio-scaled peak VO_2_ declines with age [12].

Ratio scaling is only robust if underlying mathematical assumptions are met, otherwise spurious interpretation may result [13]. Theoretically, physiological variables should be scaled for size using the general allometric equation to derive the appropriate size power function (y/x^*b*^) and thereby providing a more appropriate interpretation of size-related (x^*b*^) changes in physiologic function (y) [14]. The development of peak VO_2_ has been described using allometric scaling (log-linear analysis of covariance with body mass as the covariate) in Caucasian children and adolescents, and the same developmental pattern as absolute peak VO_2_ has been noted with age i.e., peak VO_2_ increases in boys with age, whereas in girls it increases until about 14 years of age when a leveling off is observed [15]. In a recent study, z score equations targeted to remove the effect of body size for peak VO_2_ in a group of healthy children in Canada has also been presented [16].

Peak VO_2_ has been shown to vary by ethnic group in children, with sparse data on Southern Chinese children [17, 18]. Little consideration has been given to the appropriate scaling of peak VO_2_ in the Chinese child, which is necessary for evaluating the development of peak VO_2_ and for the identification of children with low cardiopulomonary fitness. The purpose of this study therefore was to examine what the appropriate scaling approach for evaluating the development of peak VO_2_ in Southern Chinese children and adolescents should be, how power functions may differ by age and sex, and how this impacts interpretation of peak VO_2_.

## Methods

### Participants

Ethnic Chinese children and adolescents, aged 8–16 years, were recruited from the community using a stratified (by districts) and clustered (all subjects within class) random selection of primary (21 schools) and secondary schools (19 schools) from the 4 main geographical regions of Hong Kong (*i*.*e*. Hong Kong Island, Kowloon, New Territories East and New Territories West). Participation was on a voluntary basis. Informed and written consent was obtained from students and their parents. Those suffering from acute or chronic illness (for example, diabetes mellitus, kidney disease, dyslipidaemia, congenital or acquired heart disease, lupus erythematosus, sleep apnoea), or had a recent upper respiratory tract or other infection with the past 4 weeks were excluded. The study protocol was approved by the Joint Chinese University of Hong Kong—New Territory East Cluster Clinical Research Ethics Committee (CRE Ref. No. 2013.574).

### Sampling frame

The sample selection was based on a stratified (by districts) and clustered (all subjects within class) randomised sampling frame. All primary and secondary schools registered under the Education Bureau according to the four geographic regions in Hong Kong were stratified. Selection of a school was based on computer generated random numbers. If the selected school refused to participate, the next randomly selected school was invited. A total of 12 primary and 19 secondary schools were randomly chosen. Two classes in each school year grade were randomly selected from the schools. All students of the selected classes were invited to participate in the study.

### Collection of data

Participants were scheduled to undergo assessment at the cardiopulmonary exercise laboratory in the Prince of Wales Hospital. Participants were asked to abstain from alcohol, any caffeinated drink and vigorous exercise for 24 hours prior to testing. They were also asked to eat only a light snack and drink only 0.5L of water within 3 hours of the test.

### Measures

#### Anthropometric measurements

Body weight was measured to the nearest 0.1 kg and percentage body fat was measured to the nearest 0.1% using foot-to-foot bio-electrical impedance (TBF-401, Tanita Co, Tokyo, Japan) [19], with subjects barefoot and dressed in light t-shirt and shorts. Standing height without shoes was measured to the nearest 0.1 cm with a Harpenden stadiometer (Holtain, Grymych, UK). Body mass index (BMI) was calculated by weight in kg divided by height in meters squared [20].

#### Peak VO_2_

Peak VO_2_ was assessed using an incremental treadmill running test to maximum [21]. Participants began walking on the treadmill at an age-specific walking speed for 3 minutes. The speed was then increased by 1 km·h^-1^ every minute, until the speed reached the running pace of the participant. At this point the speed was held constant and the gradient increased by 1% every minute until volitional exhaustion. Heart rate was monitored continuously during the test. Participants wore a comfortably fitted facemask (Hans Rudolph paediatric large size, 8950 series) and breath-by breath gas samples were collected and analyzed throughout the test using the Medgraphics CPX/DTM metabolic cart (Medical Graphics Corporation, St. Paul, MN), calibrated prior to each test. Peak VO_2_ was determined when two of the following three conditions were reached: 1) a respiratory exchange ratio (RER)>1.0, 2) heart rate within 5% of age predicted maximum, 3) the participant was exhausted and refused to carry on despite strong verbal encouragement [22].

### Sample size calculation

Assuming peak VO_2_ is normally distributed among each age and sex, the sample size was calculated using the standard deviation of the 100α^th^ centile (SD_c100α_) and the age- and sex-specific SD described by Healy [23]. In order to determine the age and sex-specific mean and standard deviation (SD) of peak VO_2_ for sample size calculation, we collected pilot data from 261 healthy children aged 9–16 years. Age groups were analysed in 2-year intervals and the required sample size for each sex and age group was calculated to obtain the 97^th^ centile, with an error of ± 3%. We assumed that children under 9 will be the same as those under 10 years of age, and therefore proposed recruiting an additional 43 boys and 43 girls aged 8 years to help recruitment of the younger years. The estimated total number of subjects required was 828 (Table 1).

**Table 1:** Peak VO_2_ (ml·min^-1^·kg^-1^) collected from 261 healthy children aged between 9–16 years.

	Age (years)	N	Mean	SD	N required to obtain the 97^th^ centile with 3% error
Girls	9–10	24	35.3	5.3	43
	11–12	49	32.8	4.9	42
	13–14	43	31.2	5.4	52
	15–16	26	29.4	5.0	51
Boys	9–10	24	36.4	5.5	43
	11–12	50	36.9	6.8	57
	13–14	35	38.4	5.9	43
	15–16	10	42.1	6.1	40
				Total	(Sum of the above x 2 to account for each age + 86 children aged 8 year olds =) 828

### Allometric scaling

Peak VO_2_ was allometrically scaled using the procedure described by Vanderburgh et al [24, 25]. Peak VO_2_ and body weight were log-transformed. A log-linear regression model was constructed using log (peak VO_2_) as the dependent and log (body weight) as independent variables. The interaction effects of age and sex were tested and found to significantly modulate the association between body mass and peak VO_2_, justifying the need for sex and age specific exponents. Therefore, allometric scaling was done separately for 4 different subgroups: (1) younger (aged from 8 to 11.99 years) males; (2) older (aged from 12 to 16.99 years) males; (3) younger females (aged from 8 to 11.99 years) and (4) older females (aged from 12 to 16.99 years). Outliers were identified using the least median of squares eliminator and removed from analyses [26]. Regression diagnostics were performed to ensure the models were appropriate [27]. Shapiro-Wilks normality test was used to evaluate the normality of the residuals. Homoscedasticity was assessed by plotting the standardized residuals against the standardized predicted value. The resulting beta coefficients were used as the allometric exponents. Peak VO_2_ can then be allometrically scaled using the equation:

$$allometrically\;scaled\;peak\;{VO}_{2}\;=\;\frac{unscaled\;peak\;{VO}_{2}}{{body\;mass}^{exponent}}$$

Pearson correlation analysis was used to examine the association of the scaled peak VO_2_ with body size (body mass and height) and age to verify the effectiveness of the allometric scaling approach for controlling for body size within the specific age groups.

### Development of z score equations

Given the necessity for different allometric exponents for body mass in the 4 different subgroups in this dataset, z scores for the four different subgroups were developed to allow comparisons of peak VO_2_ across age and sex.

A stepwise regression was used to evaluate the association between peak VO_2_ and body size and age. The linear (x), second-order (x^2^) and third-order (x^3^) effects of age, height and weight were tested and the resulting regression equations were used to predict peak VO_2_. Subjects with the resulting standardised predicted VO_2_ greater than 3 or less than -3 were considered as outliers and were excluded. As the variance of the residual values varied with body size and the variations are different between sexes and age groups, age- and sex-specific models were established to predict the standard deviation with body size using the method suggested by Altman [28]. First, linear regression was used to examine the association between the absolute value of the residual values and body size. Since the residual values were normally distributed, the absolute values of the residual values had a half-normal distribution. The mean of a half-normal distribution is √(2/π). The SD of the residuals can be estimated as the product of the mean of the absolute residuals and √(π/2). It follows that the predicted values from the regression of the absolute residuals on body size multiplied by √(π/2) would be the estimates of SD of the signed residual, and hence of peak VO_2_. As a result, two regression models were developed for each sex and age group, one for the prediction of peak VO_2_ and the other for the prediction of SD. Z scores were calculated using the following formula:

$$Z\;score\;=\;\frac{unscaled\;peak\;{VO}_{2}-predicted\;peak\;{VO}_{2}}{predicted\;SD}$$

The calculated z scores were evaluated for departure from a normal distribution by visual assessment of histogram and by using the Shapiro-Wilk statistic.

Pearson correlation analysis was used to examine the association of the calculated z scores with body size and age to verify the validity of the computed z scores in controlling for body size and age.

### Statistical analysis

Student’s t test, Mann-Whitney U test, and chi-square test were used for group comparisons for parametric, nonparametric, and categorical data, respectively. The interaction effects of age and gender as well as pubertal stage and gender on peak VO_2_ were assessed by two-way ANOVA. The level of significance is set at p<0.05. Bonferroni correlation was used to adjust the significance level for multiple pairwise comparisons. All statistical analyses were performed using SPSS for Windows (version 21, IBM Corp., Armonk, NY, USA).

Percentile curves for absolute peak VO_2_ (expressed in l·min^-1^) and allometrically scaled peak VO_2_ were constructed using the LMS method [29]. The LMS method using the maximum penalized likelihood has been used to perform model fitting of the anthropometric centiles for the physical parameters. The LMS method estimates the measurement centiles in terms of three age- and sex-specific cubic spline curves: the L curve (Box-Cox power to transform the data that follow a normal distribution), M curve (median), and S curve (coefficient of variation).

Children’s absolute peak VO_2_ was also compared to the predicted peak VO_2_ from Armstrong and Welsman’s regression equations generated from the data of European children [12].

## Results

A total of 1386 students were invited to join the study, of whom 281 (20%) refused to participate, 37 (3%) were excluded from the study due to their medical history, 50 (4%) were unable to attend due to their parents’ schedules, 98 (7%) could not be contacted for scheduling. Of the 920 participants who underwent CPET, 68 (7%) failed to meet the criteria for a maximal effort and were excluded. Data from 852 (410 males, 442 females) were included in the final analysis. The physical characteristics of the subjects are provided in Table 2.

**Table 2:** Characteristics of male and female participants.

	Boys (n = 410)	Girls (n = 442)	P values
Age (years)	12.5 ± 2.4	12.5 ± 2.4	0.85
Height (cm)	153.5 ± 15.4	150.4 ± 11.3	<0.001
Body mass (kg)	46.1 ± 14.1	42.6 ± 11.7	<0.001
BMI (kg/m^2^)	19.2 ± 3.7	18.5 ± 3.3	0.004
Body fat (%)	18.8 ± 7.9 (n = 289)	21.0 ± 7.6 (n = 300)	0.001
Resting heart rate (beats·min^-1^)	82 ± 14	85 ± 14	0.001
Peak heart rate (beats·min^-1^)	196 ± 10	195 ± 9	0.24
Peak VO_2_ (l·min^-1^)	2.00 ± 0.73	1.58 ± 0.44	<0.001
% of predicted* peak VO_2_	87.6 ± 19.4	87.7 ± 19.3	0.98
Peak VO_2_ (ml·kg^1^·min^-1^)	43.4 ± 8.4	37.8 ± 6.7	<0.001
Peak RER	1.12 ± 0.08	1.09 ± 0.09	<0.001

VO_2_, oxygen consumption; RER, respiratory exchange ratio

*From Armstrong and Welsman’s (1994) regression equations [12]. (Boys’ peak VO_2_ = -0.623+0.230age; Girls’ peak VO_2_ = 0.253+0.124age)

### Absolute peak VO_2_

**Fig 1:**
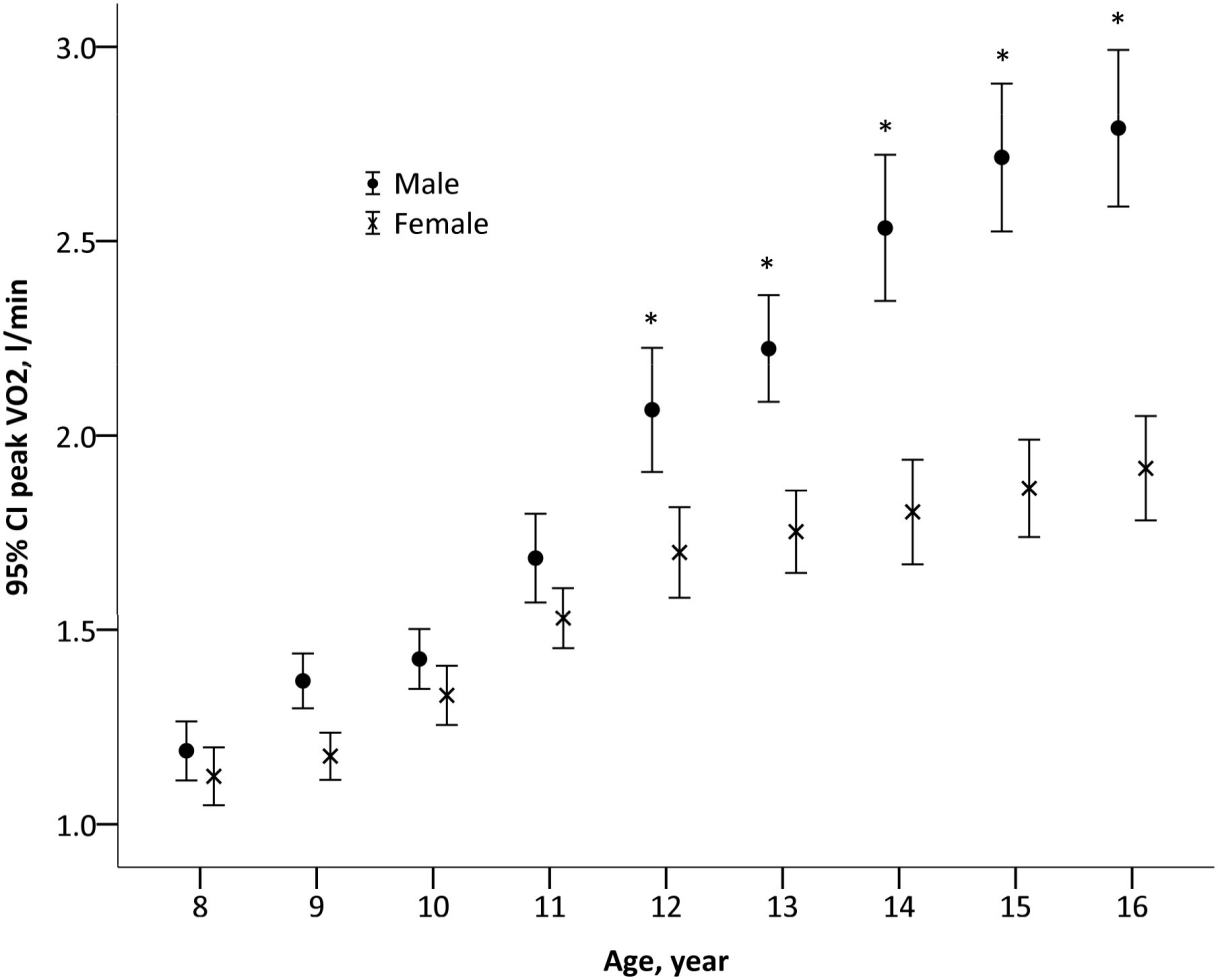
Peak VO_2_ (l·min^-1^) in boys and girls of different age groups.

**Fig 2:**
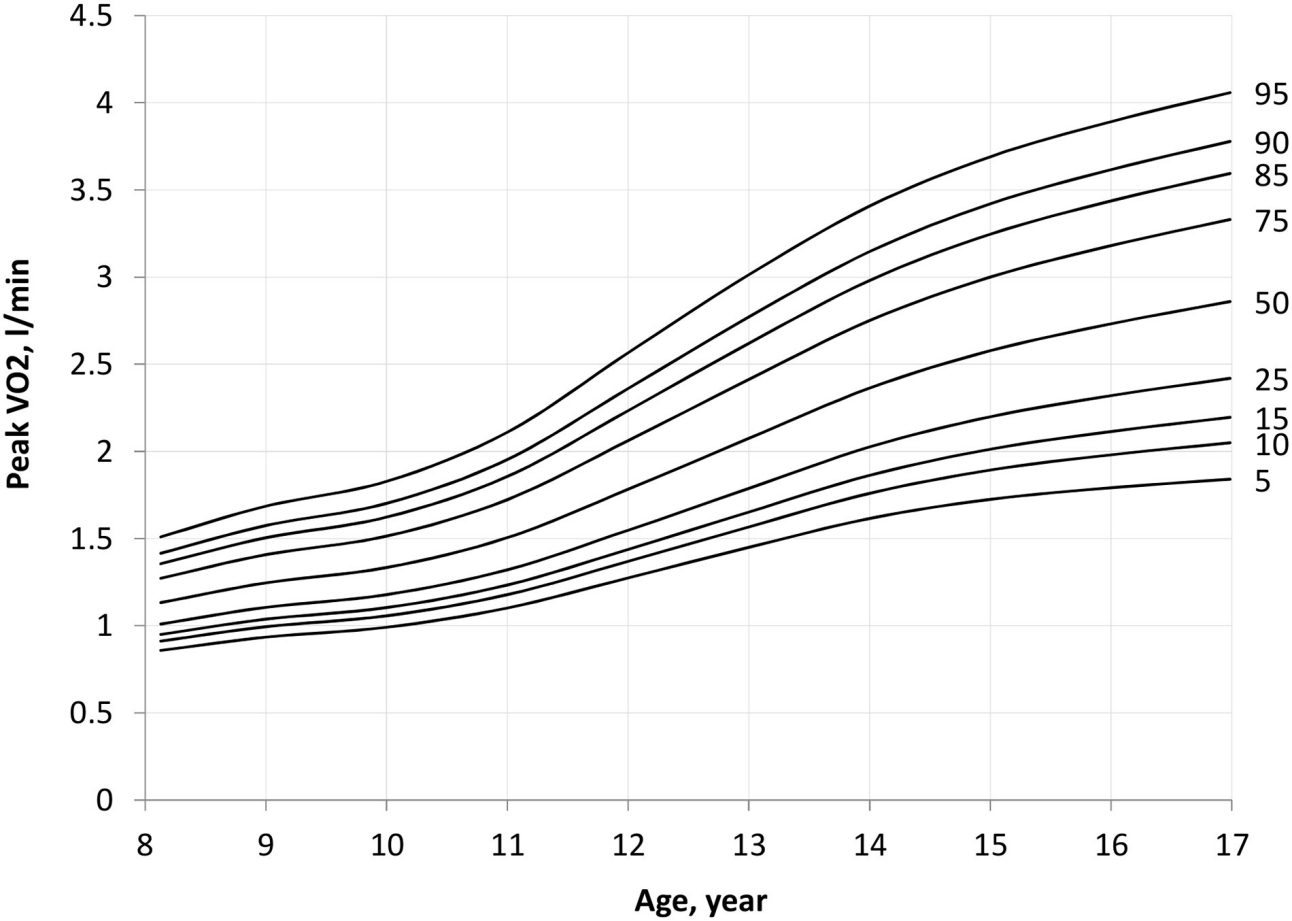
Percentile curves of peak VO_2_ (l·min^-1^) in boys.

**Fig 3:**
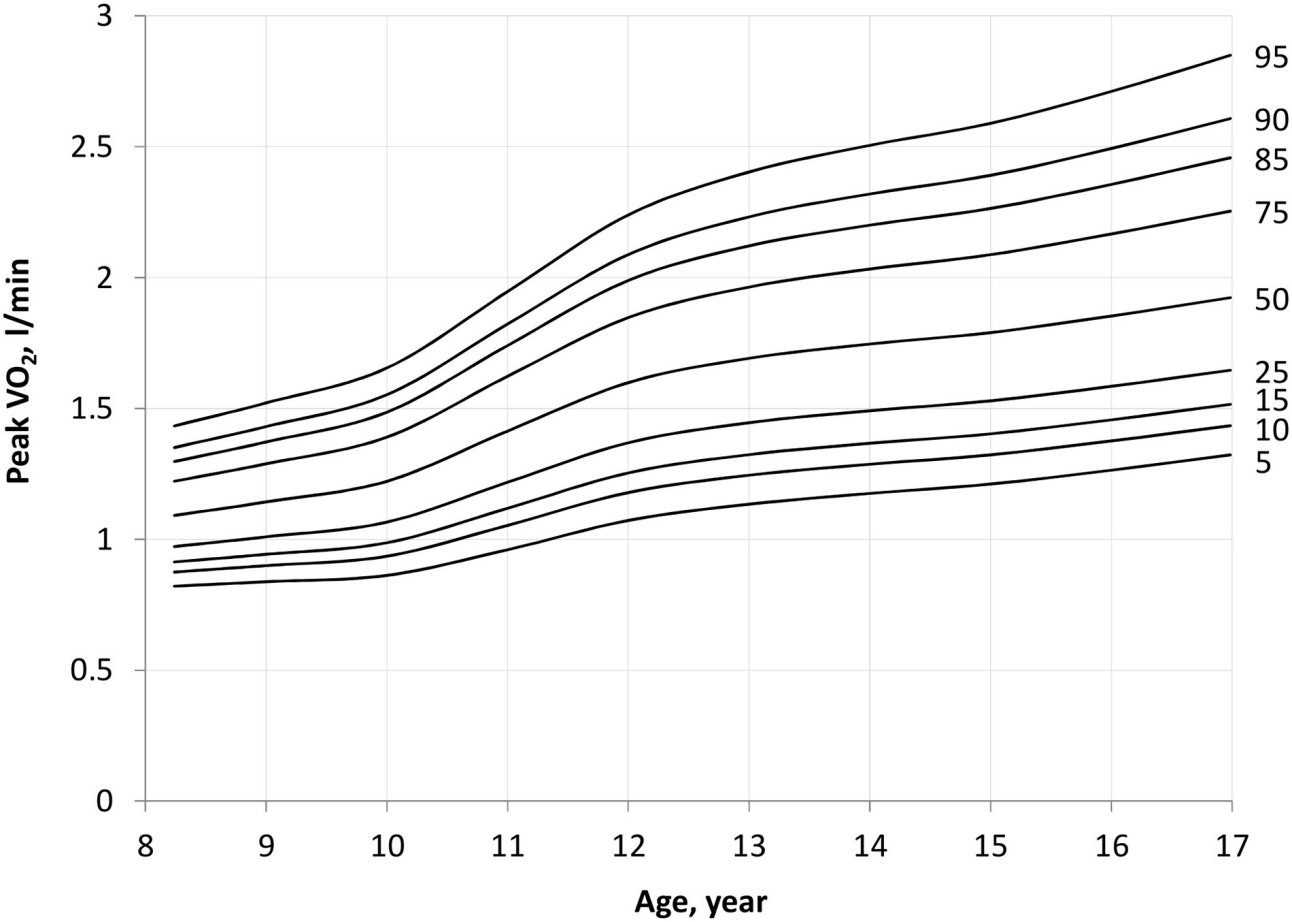
Percentile curves of peak VO_2_ (l·min^-1^) in girls.

Absolute peak VO_2_ was significantly greater in older (F_1_,_848_ = 814, *P* <0.001) and male (F_1,848_ = 52, *P* <0.001) participants. A significant age*sex interaction was present (F_1_,_834_ = 102, *P* <0.001), with subgroup analyses demonstrating an increase in peak VO_2_ with age in both males (F_1,408_ = 582, *P* <0.001) and females (F_1,440_ = 228, *P* <0.001) (Fig 1). Post-hoc analysis confirmed that the difference in peak VO_2_ between boys and girls became apparent from 12 years of age. Scatter plots of peak VO_2_ against age for boys and girls are presented in S1 and S2 Figs, respectively. Using the LMS method, smoothed percentile curves for unscaled peak VO_2_ were constructed for boys (Table 3 and Fig 2) and girls (Table 4 and Fig 3). The smoothed centiles are established with all standard errors within 9%.

**Table 3:** Smoothed centiles for peak VO_2_ (l·min^-1^) by age in boys.

Age, year	Centile (l·min^-1^)								
	5	10	15	25	50	75	85	90	95
8	0.86	0.91	0.95	1.01	1.13	1.27	1.36	1.42	1.51
9	0.93	0.99	1.04	1.11	1.25	1.41	1.50	1.57	1.69
10	0.99	1.06	1.10	1.18	1.33	1.51	1.62	1.70	1.83
11	1.10	1.18	1.23	1.32	1.51	1.72	1.86	1.95	2.11
12	1.27	1.37	1.44	1.55	1.78	2.06	2.23	2.36	2.57
13	1.45	1.57	1.65	1.79	2.07	2.41	2.62	2.77	3.01
14	1.62	1.76	1.86	2.03	2.36	2.75	2.98	3.15	3.41
15	1.72	1.89	2.01	2.20	2.58	3.00	3.25	3.42	3.69
16	1.79	1.98	2.11	2.32	2.73	3.18	3.44	3.62	3.89
Age, year	Standard error of centile (expressed as % of the centile)								
	5	10	15	25	50	75	85	90	95
8	6.4%	5.0%	4.3%	3.6%	3.2%	3.6%	4.4%	5.2%	6.8%
9	4.7%	3.8%	3.3%	2.8%	2.5%	2.9%	3.4%	4.0%	5.1%
10	4.5%	3.7%	3.3%	2.8%	2.5%	2.8%	3.4%	3.9%	5.0%
11	5.0%	4.1%	3.6%	3.1%	2.8%	3.2%	3.8%	4.4%	5.8%
12	6.0%	4.9%	4.4%	3.8%	3.4%	3.8%	4.6%	5.3%	6.8%
13	5.8%	4.6%	3.9%	3.3%	2.9%	3.3%	4.1%	4.8%	6.3%
14	7.6%	5.9%	5.0%	4.2%	3.7%	4.2%	4.9%	5.6%	7.1%
15	7.0%	5.4%	4.6%	3.8%	3.3%	3.7%	4.3%	4.8%	5.8%
16	9.0%	6.5%	5.4%	4.3%	3.7%	4.1%	4.8%	5.4%	6.6%

**Table 4:** Smoothed centiles for peak VO_2_ (l·min^-1^) by age in girls.

Age, year	Centile (l·min^-1^)								
	5	10	15	25	50	75	85	90	95
8	0.82	0.87	0.91	0.97	1.09	1.22	1.30	1.35	1.43
9	0.84	0.90	0.94	1.01	1.14	1.29	1.37	1.43	1.52
10	0.86	0.93	0.99	1.07	1.22	1.39	1.49	1.55	1.65
11	0.96	1.05	1.12	1.22	1.41	1.62	1.74	1.82	1.95
12	1.07	1.18	1.25	1.37	1.60	1.85	1.99	2.09	2.24
13	1.13	1.24	1.32	1.44	1.69	1.96	2.12	2.23	2.40
14	1.17	1.29	1.37	1.49	1.75	2.03	2.20	2.32	2.50
15	1.21	1.32	1.40	1.53	1.79	2.09	2.26	2.39	2.59
16	1.26	1.38	1.46	1.58	1.85	2.17	2.36	2.49	2.71
Age, year	Standard error of centile (expressed as % of the centile)								
	5	10	15	25	50	75	85	90	95
8	6.0%	4.7%	4.1%	3.4%	3.0%	3.4%	4.0%	4.5%	5.6%
9	5.2%	4.1%	3.6%	3.0%	2.7%	3.0%	3.4%	3.8%	4.5%
10	5.4%	4.1%	3.5%	2.9%	2.5%	2.8%	3.2%	3.6%	4.3%
11	6.5%	4.7%	3.8%	3.0%	2.6%	2.9%	3.4%	3.8%	4.6%
12	8.7%	6.2%	5.0%	4.0%	3.4%	3.9%	4.5%	5.1%	6.3%
13	6.0%	4.6%	4.0%	3.3%	2.9%	3.2%	3.7%	4.1%	4.9%
14	7.6%	5.7%	4.8%	3.9%	3.4%	3.9%	4.5%	5.2%	6.4%
15	6.7%	5.1%	4.4%	3.6%	3.2%	3.6%	4.2%	4.8%	6.0%
16	8.4%	6.4%	5.5%	4.5%	3.9%	4.5%	5.4%	6.4%	8.3%

### Ratio-scaled peak VO_2_

**Fig 4:**
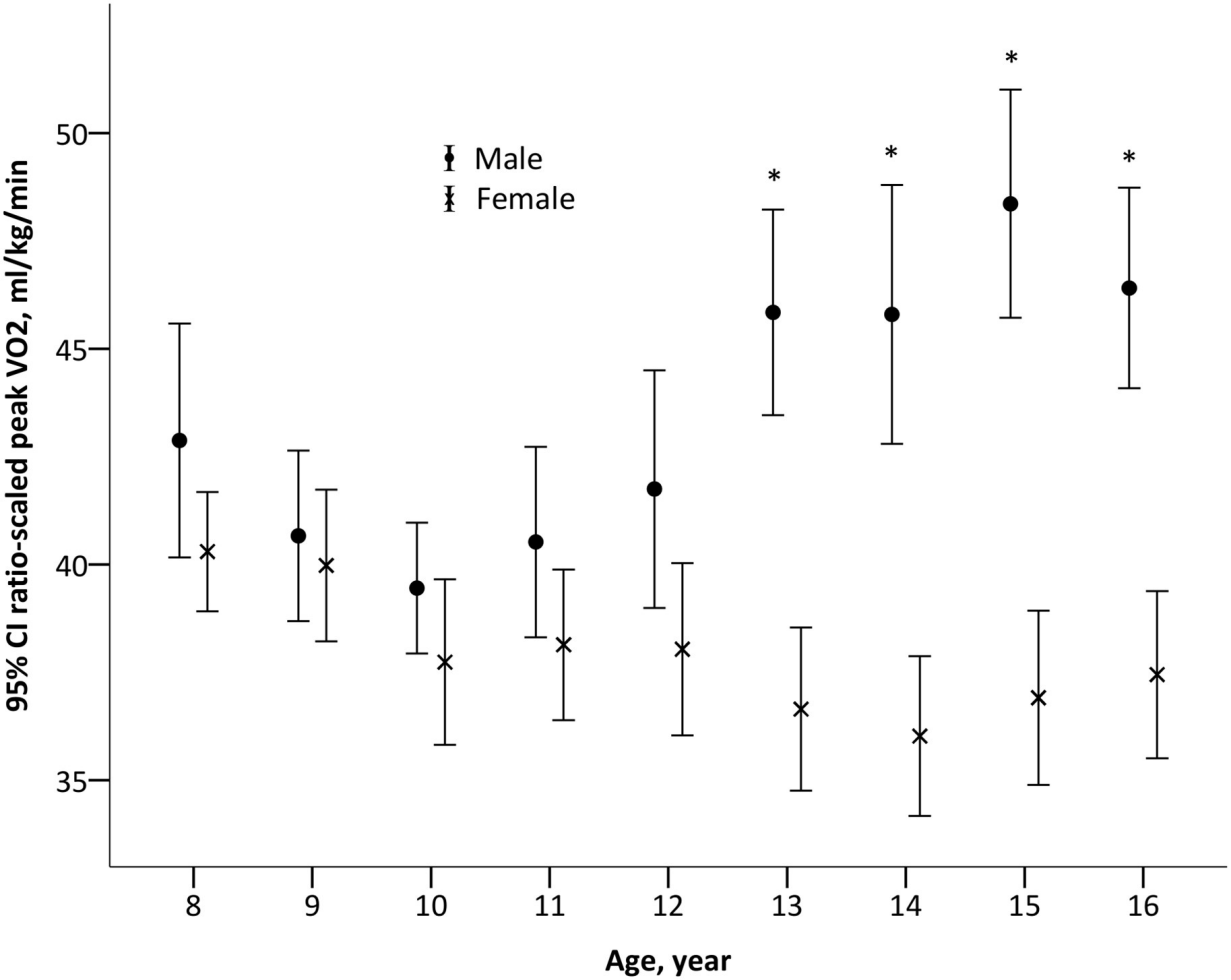
Peak VO_2_ (ml·kg^-1^·min^-1^) in boys and girls of different age groups.

When peak VO_2_ was expressed as a ratio standard with body mass (ml·kg^-1^·min^-1^), a significant main effect for age (F_1_,_848_ = 9.4, *P* = 0.002) and sex (F_1_,_848_ = 24, *P* <0.001) and a significant age*sex interaction (F_1_,_834_ = 50, *P* <0.001) were present. Ratio-scaled peak VO_2_ increased with age in boys (F_1,408_ = 43, *P* <0.001), but decreased with age in girls (F_1,440_ = 10, *P* = 0.002) (Fig 4). Differences between boys and girls were only significant from age 13 years and older.

### Allometric scaling

**Fig 5:**
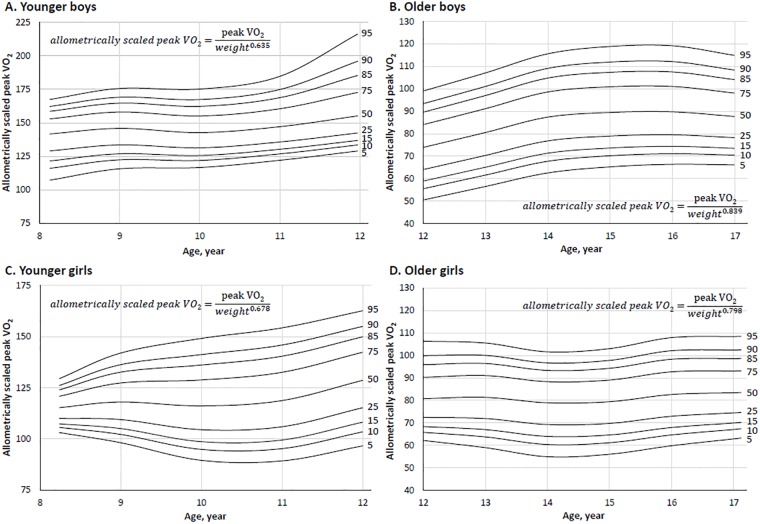
Percentile curves of allometric scaled peak VO_2_ in age- and sex-specific subgroups.

The allometric exponents generated for the younger (aged from 8 to 11.99 years) males and older (aged from 12 to 16.99 years) males were 0.635 and 0.839 respectively. For the younger females (aged from 8 to 11.99 years) and older females (aged from 12 to 16.99 years) exponents were 0.678 and 0.798. Regression diagnostics confirmed that the residuals for the log-linear regression using body mass were normally distributed, and the residuals were homoscedastic. Percentiles for the 4 subgroups are presented in Table 5 and the smoothed percentile curves for allometric scaled peak VO_2_ of the 4 subgroups are presented in Fig 5.

**Table 5:** Allometrically scaled peak VO_2_ (kg^-exponent^) by sex and age.

Sex	Male										Female								
Age	8	9	10	11	12	13	14	15	16	8	9	10	11	12	13	14	15	16
n	28	51	55	51	40	58	38	48	40	31	45	62	65	42	61	45	53	35
Exponent for body masst	0.635				0.839						0.678				0.798				
Mean	145.3	146.0	145.4	156.6	77.9	85.3	87.1	92.2	89.4	117.2	119.2	118.1	125.0	81.8	80.6	79.3	81.5	83.0
SD	16.0	19.6	16.9	25.3	15.2	16.4	17.0	17.6	14.1	11.4	14.2	21.2	18.8	13.1	14.9	13.7	16.1	13.0
5th	115.6	114.7	117.3	124.7	52.3	60.6	59.3	64.7	67.3	100.7	97.8	82.5	94.0	64.3	54.5	57.4	58.4	61.4
10th	121.9	122.3	125.4	129.6	57.2	64.0	61.8	70.0	68.4	103.8	101.6	89.1	101.1	66.9	58.1	59.3	60.3	65.4
15th	133.7	124.3	128.5	131.3	60.5	66.5	68.8	76.3	74.2	104.1	104.3	99.5	105.4	69.0	61.6	62.2	62.7	68.4
25th	136.5	133.1	133.6	141.1	66.2	70.4	75.0	80.6	79.7	109.4	108.1	105.1	109.6	72.8	70.7	68.9	69.4	73.4
50th	143.2	146.8	144.6	150.9	76.8	86.9	84.2	89.1	87.5	115.8	117.8	115.8	126.3	78.4	81.3	81.2	79.9	84.0
75th	156.6	159.3	156.9	163.7	91.3	97.5	100.0	104.0	101.3	124.3	127.3	128.2	136.2	89.5	91.0	89.4	93.7	92.0
85th	166.0	168.7	163.1	186.5	95.5	104.9	105.8	110.5	105.7	130.3	135.1	141.5	140.7	96.3	99.1	91.9	102.4	97.3
90th	171.1	171.9	166.7	199.5	97.6	108.0	110.7	116.5	113.0	135.7	138.4	149.1	147.1	103.1	100.8	96.8	105.0	97.9
95th	176.3	179.5	174.4	216.4	99.8	113.7	120.3	128.7	115.1	140.6	145.3	157.6	162.8	109.5	101.8	106.2	110.8	109.8

Allometrically scaled peak VO_2_ = peak VO_2_ / body mass^exponent^

### Z score equations

The regression equations of predicted peak VO_2_ and SD for the four different subgroups are displayed in Table 6. An automated excel file (S1 File) has been developed to calculate the z score for peak VO_2_. The resulting z score for peak VO_2_ was normally distributed and had no residual correlation with age, weight and height. The agreement between the percentile ranks from z score and allometric scaling is high (ICC = 0.95).

**Table 6:** Regression equations of predicted peak VO_2_ and SD for z score calculations.

		Adjusted R^2^	Beta coefficients for predicted mean					Beta coefficients for predicted SD		
			Height^2^ (β_1_)	Height (β_2_)	Weight (β_3_)	Age (β_4_)	Constant (β_5_)	Height (β_6_)	Age (β_7_)	Constant (β_8_)
Male	8–11.99y	0.72		10.454	15.651		-608.264	2.979		-287.994
	12–16.99y	0.49	0.057		24.418	59.533	-1300.03	7.512		-877.614
Female	8–11.99y	0.61	0.043		15.042		-57.825		31.411	-172.058
	12–16.99y	0.40			29.669		346.683	6.175		-716.619

Height is in centimeter, weight is in kilograms, and age is in year.

Regression equations for predicted peak VO_2_ = β_1_ (Height^2^) + β_2_ (Height) + β_3_ (Weight) + β_4_ (Age) + β_5_

Regression equations for predicted SD = [β_6_ (Height) + β_7_ (Age) + β_8_] × √(π/2)

Pearson correlations of absolute, ratio scaled, allometric scaled peak VO_2_, and z score of peak VO_2_ with body mass, height and age within each subgroup are presented in Table 7. All correlations between z score of peak VO_2_ and body mass, height, and age were close to zero (p>0.2 for all) in all subgroups. For allometric scaled peak VO_2_, only the correlations with body mass were close to zero (p>0.7 for all) in all subgroups. Significant associations with height and age were apparent in males and in the younger females. Both absolute and ratio-scaled peak VO_2_ were significantly associated with body size and/or age in all subgroups.

**Table 7:** Pearson correlation coefficients of unscaled and scaled peak VO_2_ with body size and age.

	Male		Female	
	8–11.99y	12–16.99y	8–11.99y	12–16.99y
Unscaled peak VO_2_				
Body mass	0.781***	0.626***	0.747***	0.636***
Height	0.763***	0.576***	0.725***	0.424***
Age	0.482***	0.461***	0.516***	0.179**
Ratio-scaled peak VO_2_ by body mass				
Body mass	-0.578***	-0.158*	-0.479***	-0.191**
Height	-0.265***	0.163*	-0.247***	-0.041
Age	-0.129	0.194**	-0.127	-0.030
Allometrically scaled peak VO_2_ (adjusted for weight using different exponents for each sex and age group)				
Body mass	-0.012	0.001	-0.019	0.007
Height	0.253***	0.266***	0.166*	0.084
Age	0.196**	0.265***	0.157*	0.028
Peak VO_2_ z score				
Body mass	0.014	-0.024	0.009	-0.003
Height	<0.001	-0.011	-0.011	0.085
Age	-0.010	-0.012	-0.003	0.051

*p<0.05,

**p<0.01,

***p<0.001

## Discussion

Our findings demonstrate that allometric scaling of peak VO_2_ for body mass was effective in removing the influence of body mass on peak VO_2_. The scaling exponent however differed by age and sex and the scaled peak VO_2_ remained correlated with height, and age, within all age- and sex-specific groups, except older girls. As a result, z score equations for different sex and age groups were developed, which were effective in removing the influence of body mass, height and age on peak VO_2_. These provide an effective metric for identifying children with low peak VO_2_.

Similar to previous reports, we show that absolute peak VO_2_ increases with age in both sexes [30–33]. A greater peak VO_2_ in boys compared to girls becomes significant from 12 to 13 years of age, with the difference gradually widening as age increases. This sex-related variability in peak VO_2_ likely relates in part to differences in body composition with boys having a higher ratio of fat-free mass/stature^2^ and a lower ratio of total body fat/stature^2^ after adolescence [34]. Indeed, we show that boys had a significantly lower percentage of body fat (thus a higher percentage of fat-free mass) than girls. In addition, the lower peak VO_2_ in the girls in our study compared to boys after 12 years of age, may be related to the onset of menarche at around 12 years in girls from Southern China [35]. which results in increases in fat-mass and a reduced rate of growth [36].

The absolute peak VO_2_ values presented here are considerably lower than the predicted values using Armstrong and Welsman’s regression equations [12], which were generated from data on Caucasian children and adolescents. It is possible that because Southern Chinese children reach peak height velocity at an earlier age, this results in less time for prepubertal growth [37] compared to Caucasian children and therefore developmentally divergent peak VO_2_. The developmental pattern of ratio-scaled peak VO_2_ in girls in the current study shows, similar to Northern Chinese girls, peak VO_2_ declined with age [38]. However, a different developmental pattern to the Northern Chinese for ratio scaled peak VO_2_ was observed in the boys in our study, with values remaining steady from 8 to 12 years, then gradually increasing. In Northern Chinese boys ratio-scaled peak VO_2_ increased from 10 to 13 years and then remained steady [38]. Despite ratio-scaled peak VO_2_ remaining the most popular method of expressing peak VO_2_ [12, 13, 39], ratio-scaled peak VO_2_ was negatively correlated with body mass and height.

When peak VO_2_ was allometrically scaled [24, 25], Pearson correlation analyses confirming that the allometric scaling effectively removed the influence of body mass. It should be noted that the exponents for our younger males and females subgroups were very close to the theoretical value of 0.67 [40], whereas in the older males and females subgroups, greater exponents were generated. These findings suggest that the influence body mass has on peak VO_2_ differs by age. The developmental pattern for allometric scaled peak VO_2_ differed from that of absolute peak VO_2_, with increases observed from 11 to 12 years of age in both sexes, and continued increases from 12 to 15 years of age in older males. Allometric scaling of peak VO_2_ is a preferred approach for the removal of body mass [41], however our data show that peak VO_2_ remains associated with height and age in males and in the younger females.

Since 4 different subgroups with different scaling exponents for body mass were required, z score calculation was developed to allows comparisons across different age and sex groups [42], which improve the ability to monitor changes in cardiopulmonary fitness longitudinally. There were no associations between z score of peak VO_2_ with body mass, height, or age in all subgroups, suggesting that the z score calculation was effective in removing the influence of body size and age on peak VO_2_.

Cardiopulmonary fitness is one of the most important predictors of lower cardiovascular and metabolic health risk and a valuable clinical diagnostic and prognostic tool. Identification of children who have relatively low cardiopulmonary function is important given improvements in cardiopulmonary function are possible with proper exercise training [43].

Both the allometric scaling and z score approaches likely provide superior decision making with respect to the identification of children with low cardiopulmonary fitness (e.g. with a percentile rank <10). It should be noted that the relatively low R-squared values (<0.5, Table 6) of the regression equations of predicted peak VO_2_ for older children (12–16.99y) may reduce the reliability of the resultant z scores [44, 45], our further analysis found that the agreement between allometric scaling and z score was high, and since the z score is independent of body size and age, it thus allows for longitudinal tracking, and may be a better choice for monitoring children and adolescents over time. Nevertheless, these are complex approaches for data treatment, and use in the clinical setting may be limited until accessible data processing is made available, and we therefore provide an excel file for easy calculation of peak VO_2_ z scores. The development of mobile apps or online sites would be important in the future for easy access to pediatric normal values.

### Study limitations

The sample size estimation was based on establishing a representative cohort with precise extreme centiles for each age and sex. The estimated sample size did not provide sufficient power for log-linear regression analysis to determine sex-specific exponents with high precision for each chronological year. Therefore, we formed younger (<12 years) and older (≥12 years) groups to ensure the precision of the exponents.

We do not include lean body mass in the current study because we only had bioelectrical impedance data on a limited number of our participants. Although scaling for lean body mass is preferred [41], many clinical testing settings like our do not have easy more valid measures of lean body mass such as dual-energy x-ray absorptiometry or magnetic resonance imaging. Besides, physical activity level was also not measured in this study so that its impact on peak VO_2_ could not be examined. In this study, the cardiopulmonary exercise test was performed on a treadmill and the reference values would not be applicable if the test was performed using other ergometers such as a cycle ergometer.

## Conclusions

In conclusion, we have provided comparisons between different body size scaling approaches for peak VO_2_ in Southern Chinese girls and boys and have shown these differentially impact the interpretation of peak VO_2_ with changes in age and sex. We recommend the use of z scores for identifying Southern Chinese children with poor cardiopulmonary fitness, and provide an accessible data processing tools for this purpose.

## Supporting information

S1 Fig Scatterplots relating VO_2_ to age for boys. (TIF)

S2 Fig Scatterplots relating VO2 to age for girls. (TIF)

S1 File Calculator for z score of peak VO_2_. (XLSX)

## References

[1] Carnethon MR, Gulati M, Greenland P. Prevalence and cardiovascular disease correlates of low cardiorespiratory fitness in adolescents and adults. Jama. 2005;294(23):2981–8. Epub 2006/01/18. 10.1001/jama.294.23.2981 . 16414945

[2] Ortega FB, Ruiz JR, Castillo MJ, Sjostrom M. Physical fitness in childhood and adolescence: a powerful marker of health. International journal of obesity (2005). 2008;32(1):1–11. Epub 2007/11/29. 10.1038/sj.ijo.0803774 . 18043605

[3] Welk GJ, Laurson KR, Eisenmann JC, Cureton KJ. Development of youth aerobic-capacity standards using receiver operating characteristic curves. American journal of preventive medicine. 2011;41(4 Suppl 2):S111–6. Epub 2011/10/14. 10.1016/j.amepre.2011.07.007 . 21961610

[4] Charlton R, Gravenor MB, Rees A, Knox G, Hill R, Rahman MA, et al Factors associated with low fitness in adolescents—A mixed methods study. BMC public health. 2014;14(1):764 10.1186/1471-2458-14-764 25074589 PMC4132898

[5] Blair SN, Kohl HW 3rd, Paffenbarger RS Jr., Clark DG, Cooper KH, Gibbons LW. Physical fitness and all-cause mortality. A prospective study of healthy men and women. Jama. 1989;262(17):2395–401. Epub 1989/11/03. . 2795824 10.1001/jama.262.17.2395

[6] Guimaraes GV, d’Avila VM, Camargo PR, Moreira LF, Lanz JR, Bocchi EA. Prognostic value of cardiopulmonary exercise testing in children with heart failure secondary to idiopathic dilated cardiomyopathy in a non-beta-blocker therapy setting. European journal of heart failure. 2008;10(6):560–5. Epub 2008/05/20. 10.1016/j.ejheart.2008.04.009 . 18486551

[7] Nixon PA, Orenstein DM, Kelsey SF, Doershuk CF. The prognostic value of exercise testing in patients with cystic fibrosis. N Engl J Med. 1992;327(25):1785–8. Epub 1992/12/17. 10.1056/NEJM199212173272504 . 1435933

[8] Pianosi P, Leblanc J, Almudevar A. Peak oxygen uptake and mortality in children with cystic fibrosis. Thorax. 2005;60(1):50–4. Epub 2004/12/25. 10.1136/thx.2003.008102 . 15618583 PMC1747160

[9] Paridon SM. Congenital Heart Disease: Cardiac Performance and Adaptations to Exercise. Pediatric exercise science. 1997;9(4):308–23. 10.1123/pes.9.4.308

[10] Cunningham DA, Paterson DH, Blimkie CJR. The development of the cardiorespiratory system with growth and physical activity In: Boileau RA, editor. Advances in Pediatric Sport Sciences 1. Champaign IL: Human Kinetics; 1984 p. 85–116.

[11] Rowland TW. Developmental aspects of physiological function relating to aerobic exercise in children. Sports medicine (Auckland, NZ). 1990;10(4):255–66. Epub 1990/10/01. 10.2165/00007256-199010040-00004 . 2247726

[12] Armstrong N, Welsman JR. Assessment and interpretation of aerobic fitness in children and adolescents. Exercise and sport sciences reviews. 1994;22:435–76. Epub 1994/01/01. . 7925551

[13] Tanner JM. Fallacy of per-weight and per-surface area standards, and their relation to spurious correlation. Journal of applied physiology. 1949;2(1):1–15. Epub 1949/07/01. 10.1152/jappl.1949.2.1.1 . 18133122

[14] Schmidt-Nielsen K. Scaling: Why is animal size so important?. Cambridge: Cambridge University Press; 1984 21–32 p.

[15] Welsman JR, Armstrong N, Nevill AM, Winter EM, Kirby BJ. Scaling peak VO2 for differences in body size. Medicine and science in sports and exercise. 1996;28(2):259–65. Epub 1996/02/01. . 8775163 10.1097/00005768-199602000-00016

[16] Blanchard J, Blais S, Chetaille P, Bisson M, Counil FP, Huard-Girard T, et al New Reference Values for Cardiopulmonary Exercise Testing in Children. Medicine and science in sports and exercise. 2018;50(6):1125–33. Epub 2018/01/19. 10.1249/MSS.0000000000001559 . 29346167 PMC6023574

[17] McManus AM, Chung Yung T, Leung MP. Peak oxygen uptake in relation to age, sex, and maturation in Hong Kong Chinese children. American journal of human biology: the official journal of the Human Biology Council. 2004;16(5):602–5. Epub 2004/09/16. 10.1002/ajhb.20061 . 15368609

[18] Sheng LW, Ye JC, Qing ZY, M ZIN, Xin SL, Jie GM. Maximal aerobic power in children and adolescents of Beijing, China. American journal of human biology: the official journal of the Human Biology Council. 1996;8(4):497–503. Epub 1996/01/01. 10.1002/(sici)1520-6300(1996)8:4<497::Aid-ajhb10>3.0.Co;2-h . 28557075

[19] Sung RY, So HK, Choi KC, Li AM, Yin J, Nelson EA. Body fat measured by bioelectrical impedance in Hong Kong Chinese children. Hong Kong medical journal = Xianggang yi xue za zhi. 2009;15(2):110–7. Epub 2009/04/04. . 19342736

[20] So HK, Nelson EA, Li AM, Wong EM, Lau JT, Guldan GS, et al Secular changes in height, weight and body mass index in Hong Kong Children. BMC public health. 2008;8:320 Epub 2008/09/23. 10.1186/1471-2458-8-320 . 18803873 PMC2572616

[21] Yu CCW, McManus AM, Li AM, Sung RYT, Armstrong N. Cardiopulmonary exercise testing in children. HK J Paediatr (New Series). 2010;15:13. Epub 47.

[22] Yu CC, Li AM, So RC, McManus A, Ng PC, Chu W, et al Longer term follow up of aerobic capacity in children affected by severe acute respiratory syndrome (SARS). Thorax. 2006;61(3):240–6. Epub 2006/02/02. 10.1136/thx.2005.046854 . 16449271 PMC2080724

[23] Healy HJ. Statistics of growth standards In: Falkner F, Tanner JM, editors. Human growth: a comprehensive treatise: methodology, ecological, genetic and nutritional effects on growth. New York: Plenum Press; 1986 p. 47–58.

[24] Vanderburgh PM, Mahar MT, Chou CH. Allometric scaling of grip strength by body mass in college-age men and women. Research quarterly for exercise and sport. 1995;66(1):80–4. Epub 1995/03/01. 10.1080/02701367.1995.10607658 . 7777699

[25] Vanderburgh PM, Katch FI, Schoenleber J, Balabinis CP, Elliott R. Multivariate allometric scaling of men’s world indoor rowing championship performance. Medicine and science in sports and exercise. 1996;28(5):626–30. Epub 1996/05/01. . 9148095 10.1097/00005768-199605000-00015

[26] Blatná D. Outliers in regression. Trutnov. 2006;30:1–6.

[27] Batterham AM, George KP. Allometric modeling does not determine a dimensionless power function ratio for maximal muscular function. Journal of applied physiology (Bethesda, Md: 1985). 1997;83(6):2158–66. Epub 1998/02/14. 10.1152/jappl.1997.83.6.2158 . 9390994

[28] Altman DG. Construction of age‐related reference centiles using absolute residuals. Statistics in medicine. 1993;12(10):917–24. 8337548 10.1002/sim.4780121003

[29] Cole TJ. The LMS method for constructing normalized growth standards. European journal of clinical nutrition. 1990;44(1):45–60. Epub 1990/01/01. . 2354692

[30] Armstrong N, Williams J, Balding J, Gentle P, Kirby B. The peak oxygen uptake of British children with reference to age, sex and sexual maturity. European journal of applied physiology and occupational physiology. 1991;62(5):369–75. Epub 1991/01/01. . 1874245 10.1007/BF00634975

[31] Krahenbuhl GS, Skinner JS, Kohrt WM. Developmental aspects of maximal aerobic power in children. Exercise and sport sciences reviews. 1985;13:503–38. Epub 1985/01/01. . 3891374

[32] Nes BM, Osthus IB, Welde B, Aspenes ST, Wisloff U. Peak oxygen uptake and physical activity in 13- to 18-year-olds: the Young-HUNT study. Medicine and science in sports and exercise. 2013;45(2):304–13. Epub 2012/09/13. 10.1249/MSS.0b013e318271ae4d . 22968311

[33] Rowland TW. The development of aerobic fitness in children.. London: E & FN Spon; 1997 179–90 p.

[34] Maynard LM, Wisemandle W, Roche AF, Chumlea WC, Guo SS, Siervogel RM. Childhood body composition in relation to body mass index. Pediatrics. 2001;107(2):344–50. Epub 2001/02/07. . 11158468 10.1542/peds.107.2.344

[35] Lam TH, Shi HJ, Ho LM, Stewart SM, Fan S. Timing of pubertal maturation and heterosexual behavior among Hong Kong Chinese adolescents. Archives of sexual behavior. 2002;31(4):359–66. Epub 2002/08/22. . 12187549 10.1023/a:1016228427210

[36] Karapanou O, Papadimitriou A. Determinants of menarche. Reproductive Biology and Endocrinology. 2010;8(1):115 10.1186/1477-7827-8-115 20920296 PMC2958977

[37] Floyd B. The contribution of adolescent growth to shorter adult statures among girls of Chinese ancestry. American Journal of Human Biology. 1998;10(6):735–46. 10.1002/(SICI)1520-6300(1998)10:6<735::AID-AJHB5>3.0.CO;2-I 28561407

[38] Lin WS, Ye JC, Qing ZY, M ZIN, Xin SL, Jie GM. Maximal aerobic power in children and adolescents of Beijing, China. American journal of human biology: the official journal of the Human Biology Council. 1996;8(4):497–503. Epub 1996/01/01. 10.1002/(sici)1520-6300(1996)8:4<497::aid-ajhb10>3.0.co;2-h . 28557075

[39] Poehlman ET, Toth MJ. Mathematical ratios lead to spurious conclusions regarding age- and sex-related differences in resting metabolic rate. The American journal of clinical nutrition. 1995;61(3):482–5. Epub 1995/03/01. 10.1093/ajcn/61.3.482 . 7872210

[40] Nevill AM, Ramsbottom R, Williams C. Scaling physiological measurements for individuals of different body size. European journal of applied physiology and occupational physiology. 1992;65(2):110–7. Epub 1992/01/01. . 1396632 10.1007/BF00705066

[41] Loftin M, Sothern M, Abe T, Bonis M. Expression of VO2peak in Children and Youth, with Special Reference to Allometric Scaling. Sports medicine (Auckland, NZ). 2016;46(10):1451–60. Epub 2016/05/04. 10.1007/s40279-016-0536-7 . 27139725

[42] Wang Y, Chen H-J. Use of Percentiles and Z-Scores in Anthropometry In: Preedy VR, editor. Handbook of Anthropometry: Physical Measures of Human Form in Health and Disease. New York, NY: Springer New York; 2012 p. 29–48.

[43] Matos N, Winsley RJ. Trainability of young athletes and overtraining. Journal of sports science & medicine. 2007;6(3):353–67. Epub 2007/01/01. . 24149422 PMC3787286

[44] Cantinotti M, Kutty S, Franchi E, Paterni M, Scalese M, Iervasi G, et al Pediatric echocardiographic nomograms: What has been done and what still needs to be done. Trends Cardiovasc Med 2017;27(5):336–49. 10.1016/j.tcm.2017.01.006 Epub 2017 Jan 19. 28214110

[45] Cantinotti M, Scalese M, Giordano R, Franchi E, Assanta N, Marotta M, Viacava C, Molinaro S, Iervasi G, Santoro G, Koestenberger M.Normative Data for Left and Right Ventricular Systolic Strain in Healthy Caucasian Italian Children by Two-Dimensional Speckle-Tracking Echocardiography. J Am Soc Echocardiogr 2018;31(6):712–20.e6. 10.1016/j.echo.2018.01.006 29526564

