# Supplementary figures and images for: Appropriate scaling approach for evaluating peak VO2 development in Southern Chinese 8 to 16 years old
Source: PLoS One. 2019 Mar 12;14(3):e0213674. doi: 10.1371/journal.pone.0213674 (PMC6413916; doi:10.1371/journal.pone.0213674)

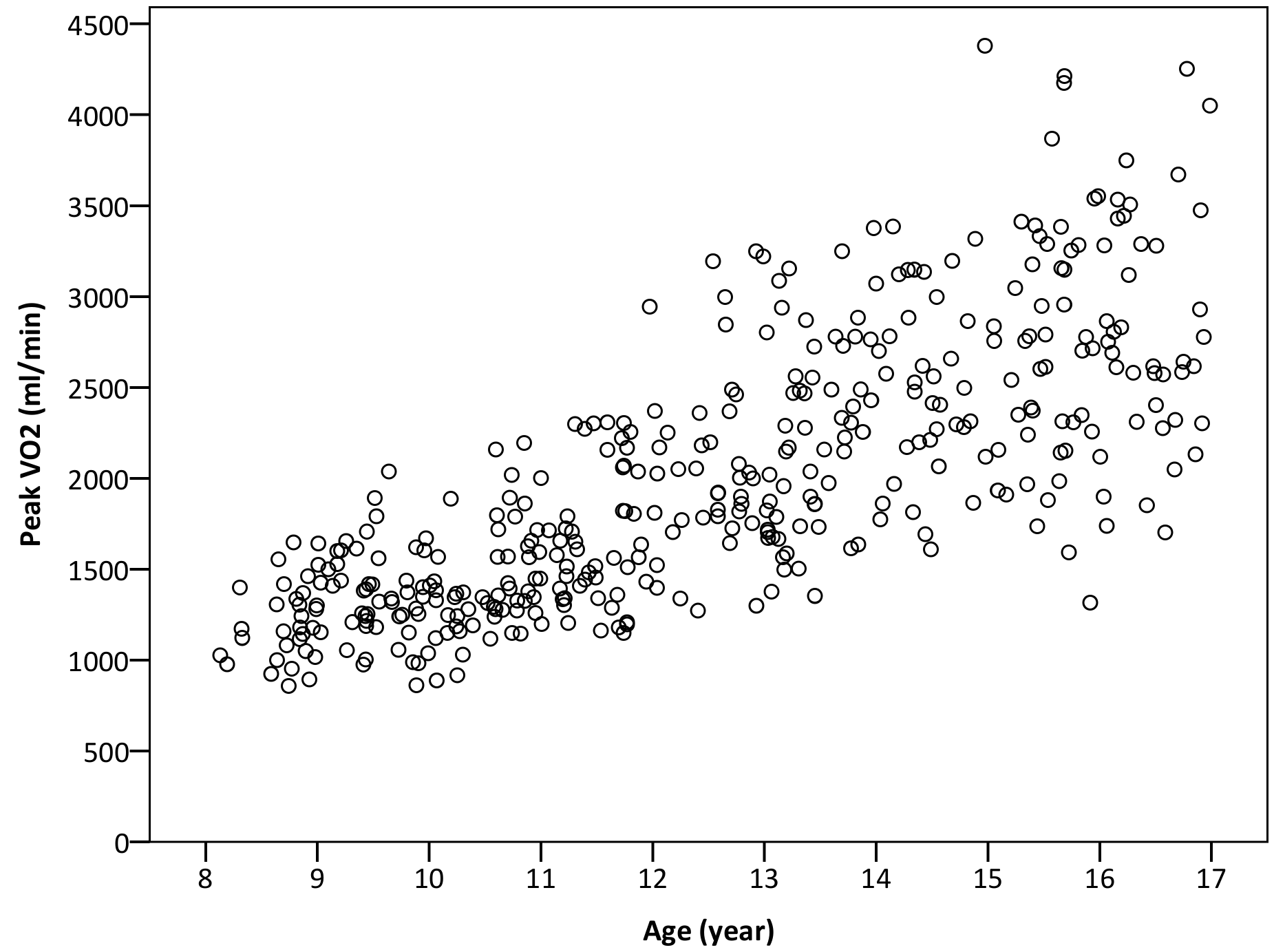

Supplement: S1 Fig — (TIF) [file pone.0213674.s001.tif]

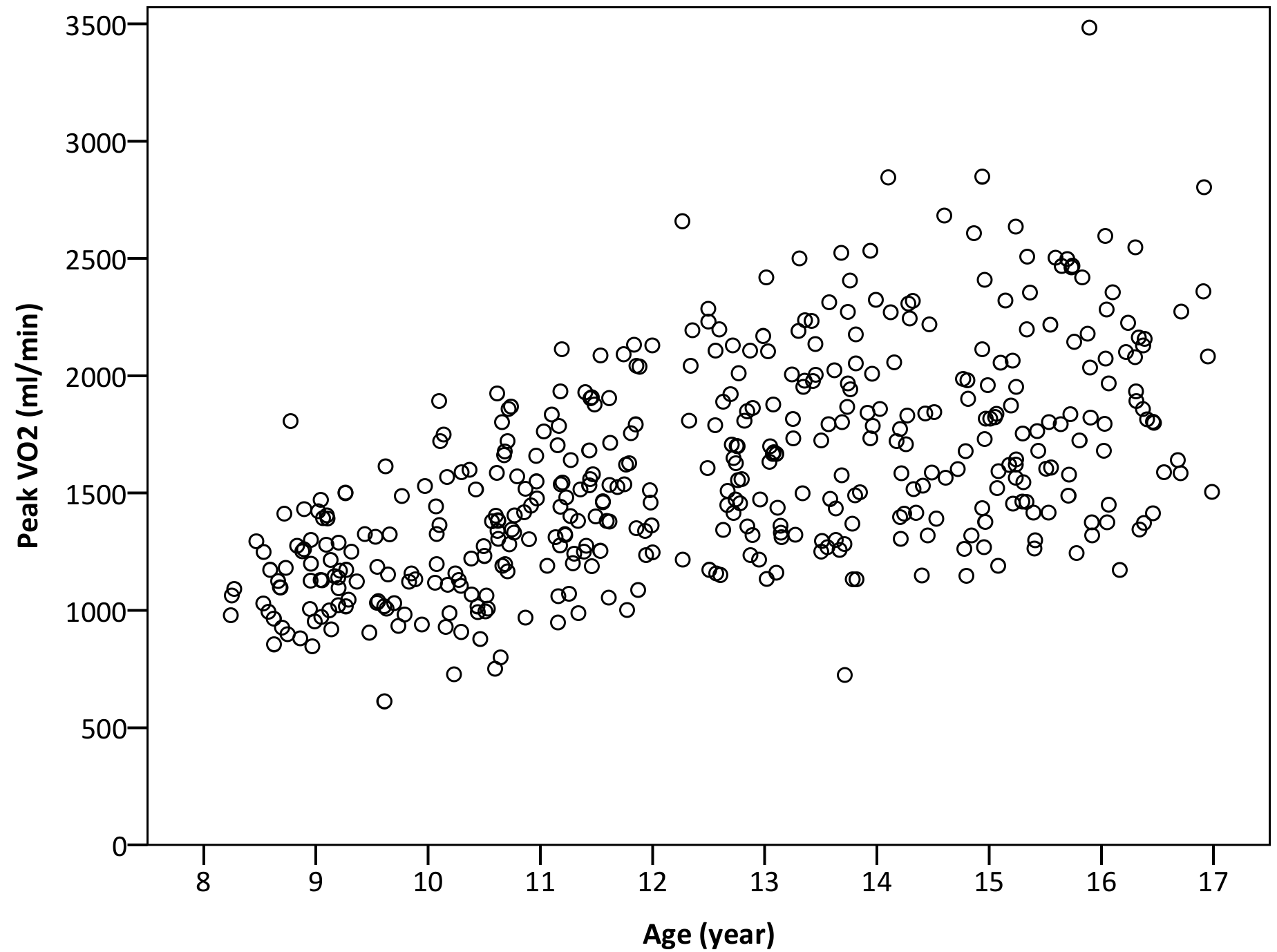

Supplement: S2 Fig — (TIF) [file pone.0213674.s002.tif]
